# Agrivoltaic arrays and effects of forage biomass and nutritive value of grasses and legumes for grazing dairy cattle

**DOI:** 10.3168/jdsc.2025-0973

**Published:** 2026-04-09

**Authors:** Sabrina L. Florentino, Eric S. Buchanan, Michael H. Reese, Andre F. Brito, Bradley J. Heins

**Affiliations:** 1 Department of Animal Science, University of Minnesota, St. Paul, MN 55108; 2 West Central Research and Outreach Center, University of Minnesota, Morris, MN 56267; 3 Department of Agriculture, Nutrition, and Food Systems, University of New Hampshire, Durham, NH 03824

## Abstract

Forage crops for grazing dairy cattle grown underneath ground-mounted photovoltaic (PV) systems may provide a feed source for livestock production. The objective was to evaluate forage biomass and nutritive value of grasses and legumes grown under different PV conditions. Forages were planted underneath a 30-kW PV site (30kW), a 50-kW PV site (50kW), and one control pasture site without PV (CON) in 2022 and 2023 with 4 replicates per site. Solar sites were all located on the University of Minnesota West Central Research and Outreach Center grazing dairy farm (Morris, MN) and were not located next to each other, but were 700 m apart. Forage crops included alfalfa (*Medicago sativa* L.), field peas (*Pisum sativum* L.), meadow fescue (*Schedonorus pratensis* (Huds.) P. Beauv), orchardgrass (*Dactylis glomerata* L.), red clover (*Trifolium pratense* L.), brown midrib sorghum-Sudangrass (*Sorghum bicolor* [L.] Moench ssp. *Drummondii* [Steud.]), white clover (*Trifolium repens* L.), and 3 grass and legume mixes with either alfalfa, red clover, or white clover. Forage samples were clipped (3 clippings per year) when the forages reached ~25 to 35 cm in height, which corresponded to recommended pregrazing height for lactating dairy cows. Samples were analyzed for forage nutritive value. Forage biomass, DM, and nutritive values were analyzed with PROC MIXED of SAS with the fixed effects of site (30kW, 50kW, or pasture), forage nested within site, year (2022 and 2023), and cutting (1, 2, or 3), and the random effect of replicate nested within site. Forages produced less biomass at the 50-kW (3,223 kg/ha) solar sites compared with 30-kW solar site (8,968 kg/ha) and the control pasture (9,987 kg/ha). The 50kW forages had greater crude protein on a DM basis (23.8%) than the 30kW (20.1%) and control pasture (18.2%). The 50kW (54.4%) forages also had greater total-tract NDF digestibility than the 30kW (52.3%) and control pasture (49.1%). Forage biomass and nutrient nutritive values varied based on the solar array design and amount of sun exposure.

## Highlights

• Forage biomass was lowest for grass species grown under the most shaded solar array (50 kW).
• Forages in AV systems had a higher crude protein content.
• Shaded forages showed higher total-tract fiber digestibility.
• Agrivoltaics produce high-quality forages for grazing livestock.

## Graphical Abstract

Agrivoltaic (AV) systems, which combine solar energy production and agriculture on the same land, present a strategy for sustainable intensification of agriculture and renewable energy generation. This study evaluated the potential of growing various grasses and legumes underneath 2 different ground-mounted photovoltaic (PV) arrays (30 and 50 kW) compared with a control (CON) pasture for grazing dairy cattle. Results show that the 50-kW PV site produced less forage biomass compared with the 30-kW site) and the CON pasture, primarily due to reduced sunlight. However, the forages grown at the 50-kW site demonstrated greater nutritive value. This suggests that AV systems, especially those with increased shading, can produce high-quality forage that can be a suitable feed source for grazing livestock, potentially offsetting lower biomass yields through increased nutritional content.

**Figure:**
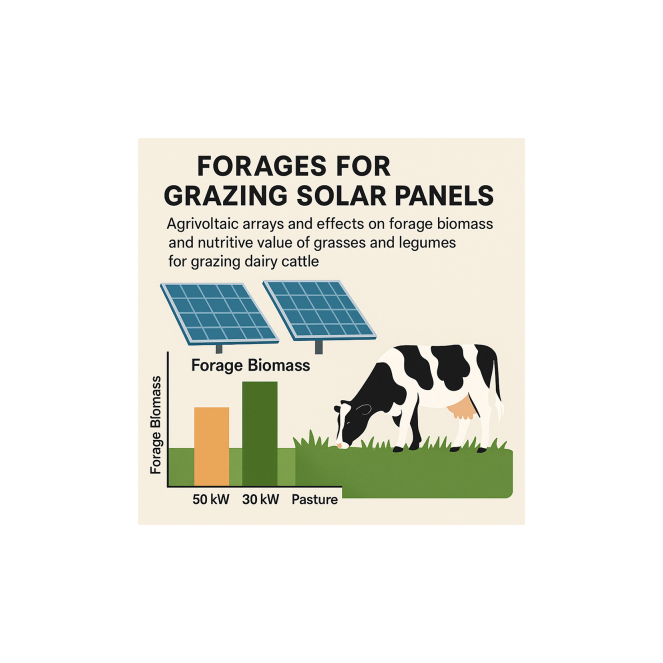

Increasing land availability pressures demand the sustainable intensification of agriculture. Solar energy sites, which take arable land out of agriculture cultivation, minimize food production potential. At the same time, climate change and population increases are decreasing available arable land (Hultgren et al., 2025). Although renewable solar energy often takes land out of production, it replaces GHG-emitting fossil fuels by generating electricity with minimal direct carbon emissions (Peng et al., 2013). The agrivoltaics (**AV**) concept has been developed to combine solar energy and agricultural production on one site, which accomplishes both food production and clean energy goals while providing flexible economic opportunities to farmers. Agrivoltaics refers to the colocation of agricultural production and photovoltaic electricity generation on the same land area to optimize land use efficiency and support food and energy production (Dupraz et al., 2011).

Agronomic conditions in grazing AV systems affect crop biomass production and nutritive value (Bacon et al., 2025). Agrivoltaics systems decrease air and soil temperature and increase soil moisture below the solar panels (Marrou et al., 2013; Barron-Gafford et al., 2019). Furthermore, AV systems do not consistently increase or decrease airflow, do not increase carbon dioxide production from soil, and may reduce soil respiration due to cooler, and more moist conditions (Barron-Gafford et al., 2019). This microclimate produces favorable conditions for crops such as winter wheat and potatoes, especially in drought years (Amaducci et al., 2018; Trommsdorff et al., 2021; Sturchio et al., 2022). Additionally, the reduction in air temperature due to the inclusion of plants for ground cover minimizes heat stress on the solar panels, increasing energy production (Adeh et al., 2019; Barron-Gafford et al., 2019; Williams et al., 2023). Forage crops grown underneath ground-mounted photovoltaic (**PV**) systems could provide a feed source and shade for grazing dairy cattle production systems, a green energy source for farms, and additional income for pasture-based farmers (Sharpe et al., 2021). However, yield response to varying levels of shade and altered microclimatic conditions for common forages grown in pastures under solar PV systems has not been studied.

Modeling suggests that AV systems could increase land productivity from 35% to 73% worldwide (Dupraz et al., 2011; Amaducci et al., 2018; Trommsdorff et al., 2021). These improvements are reflected in the land equivalent ratio (**LER**), which accounts for the combined output of agricultural production (i.e., crop biomass, livestock forage, livestock production) and photovoltaic electricity generation per unit area. Other studies record LER greater than 1.5 for both crop and livestock production in AV systems indicating synergistic benefits of solar energy and agriculture colocation (Amaducci et al., 2018; Andrew et al., 2021; Trommsdorff et al., 2021). These estimates do not account for newer spectral-splitting solar panels, which have been documented to increase plant biomass compared with grasses in legumes in pastures. This technology allows a specific range of the light spectrum useful for photosynthesis to pass through the panel, while the rest of the spectrum is reflected in energy production, increasing the efficiency of the system (Zhang et al., 2023).

Adopting AV systems reduces GHG emissions while increasing revenue for farmers (Proctor et al., 2021). For example, AV systems incorporating livestock grazing produced greater income by 2.5% to 35% compared with conventional systems (Thompson et al., 2020; Lytle et al., 2021). The AV systems may increase land efficiency and improve farm income, but further documentation of the agronomic potential of these grazing systems is needed. Therefore, the objective of this study was to evaluate the biomass production and nutritive value of forages and legumes when grown in pastures and under solar PV systems on pasture for grazing dairy production systems.

The research was conducted at the certified organic dairy research farm at the University of Minnesota West Central Research and Outreach Center (Morris, MN). All treatments were located within the same research farm under certified organic management. The study was conducted from May 2022 to September 2022 and from May 2023 to September 2023. One solar site was a 30-kW system that consisted of two 15-kW fixed solar arrays mounted at 35 degrees south and 2.5 to 3.0 m off the ground (**30kW**). The other solar site was a 50-kW, square shaped, flat-top array using reflectors mounted 2.5 to 3.0 m off the ground (**50kW**). The control treatment (**CON**) was in an adjacent pasture without an AV system and with minimal slope, adjacent to the 50kW system, and similar soil type. No cattle were allowed to graze any of the experimental plots during the study (CON or AV treatments), and all forage treatments were managed similarly with respect to tillage and establishment. The sites were located in close proximity to each other (700 m apart) on the research farm, which minimized differences in long-term management, soil type, and environmental conditions. The organic research site is certified by the USDA National Organic Program.

Because the AV systems were pre-existing infrastructure on the research farm, treatments could not be randomly assigned to identical physical locations. Although all sites were located within the same farm and shared similar soil and management history, some site-specific effects may not be completely excluded. Although the AV sites were elevated to allow livestock access, plots were not grazed during the experimental period to ensure uniform treatment conditions across sites.

The University of Minnesota West Central and Outreach Center Weather station recorded daily ambient temperature, soil temperature, and precipitation. The weather station was 170 m from 50kW, 304 m from 30kW, and 549 m from CON. The highest monthly average ambient temperature was 23.3°C in July, and the lowest was 8.9°C in October. The highest monthly total rainfall was 15.6 cm in May, and the lowest 2.3 cm in September. Overall, the ambient and soil temperatures gradually increased from May to July and August and then decreased through October, whereas rainfall varied substantially, being highest in May and generally decreasing later in the season. Average temperature from May to October was 17.6°C in 2022 and 18.6°C in 2023. Precipitation totaled 56 cm in 2022 and 65 cm in 2023 (WCROC, 2025), both of which were below the long-term normal of 68 cm.

The research sites were not fertilized before planting except for the deposition of urine and dung by grazing cattle from previous years of cattle underneath solar panels in research experiments (Sharpe et al., 2021). All sites were tilled once before establishment. During May of 2022, 7 forage species and 3 mixes of grass and legume species were planted underneath the 30kW, 50kW, and CON sites. The forages were established in 2022, and initial harvest was in 2022; however, a full production year was in 2023. Forage crops included alfalfa (*Medicago sativa* L.), field peas (*Pisum sativum* L.), meadow fescue (*Schedonorus pratensis* (Huds.) P. Beauv), orchardgrass (*Dactylis glomerata* L.), red clover (*Trifolium pratense* L.), brown midrib sorghum-Sudangrass (*Sorghum bicolor* [L.] Moench ssp. *Drummondii* [Steud.]), white clover *(Trifolium repens* L.), and 3 grass and legume mixes with either alfalfa, red clover, or white clover. The grass and legume mixes and field peas were not planted in the 50kW site because this solar array was 20% smaller in land size. Forages were planted with a Carter plot seeder (Carter Manufacturing Company Inc.) at recommended species seeding rates: alfalfa, meadow fescue, and orchardgrass at 16.8 kg/ha, field peas at 89.7 kg/ha, red clover at 13.5 kg/ha, sorghum-Sudangrass at 22.4 kg/ha, and white clover at 9.0 kg/ha. Each species of the 3 grass and legume mixes was seeded at the same rate as when planted in monoculture. This approach was chosen to ensure adequate establishment of both grass and legumes under organic conditions and to evaluate species performance without reducing individual seeding rates. All crops were managed on certified organic pastureland. The experimental design was a randomized complete block design within each site. Four replicate rows served as blocks to account for potential spatial variation in soil characteristics and light distribution across the site. Within each row, the 10 forage treatments were randomly assigned to individual plots. Individual plots measured 5.5 m × 1.8 m.

Two biomass samples from 3 cuttings each year (2022 and 2023) were hand-clipped of each forage from each plot using a 0.23 m^2^ square. Forage samples were clipped at vegetative growth stages consistent with recommended pregrazing management for dairy cattle. Cool-season grasses and legumes were harvested at 25 to 35 cm. However, sorghum-Sudangrass was harvested at 120 to 150 cm, which corresponded to recommended vegetative grazing stages (Ritz et al., 2020, 2021). The entire plot was mowed to 10-cm stubble height after biomass sampling with a Jari plot sickle mower (Jari Mowers, Mankato, MN) to mimic cattle grazing. A target post-cut residual height of 10 cm was used across all treatments to standardize regrowth potential and minimize variability in grazing simulation. All forages except the field peas were sampled 3 times during the growing season. Samples were dried at 60°C for 99 h to determine DM concentration. One of the 2 samples from each plot was randomly selected and sorted for botanical composition. Once sorted, samples were ground with a 1-mm screen (Model 4, Wiley Mill, Thomas Scientific, Swedesboro, NJ) and stored in plastic bags until they were shipped for nutritive analysis. Samples were analyzed for forage nutritive value at Rock River Laboratory Inc. (Watertown, WI). Crude protein (AOAC Method 990.03; AOAC International, 2023b), NDF (AOAC Method 2002.04; AOAC International, 2023c), and ADF (AOAC Method 973.18; AOAC International, 2023a) were determined with near-infrared reflectance spectroscopy calibrated against wet chemistry methods (Mertens, 2022). Mineral concentrations were analyzed using inductively coupled plasma spectroscopy (AOAC Official Method 2015.01; AOAC International, 2023d). Total-tract NDF digestibility (**TTNDFD**) was determined with validated in vitro procedures (Lopes et al., 2015).

Forage biomass, DM concentration, and nutritive value parameters were analyzed using the PROC MIXED procedure of SAS (version 9.4, SAS Institute Inc., Cary, NC; SAS Institute, 2024). The experimental design was a randomized complete block design within site, and replicate was a random effect. Fixed effects included site (30kW, 50kW, or CON), year (2022 or 2023), cutting (1, 2, or 3), and forage species nested within site. Replicate nested within site was included as a random effect. Significance was declared at *P* < 0.05. Year was included as a fixed effect in the model, but no significant year by site interactions were observed (*P* > 0.05), and therefore data were presented as averages across years.

The first cutting for all forages had more biomass (*P* < 0.05) compared with cuttings 2 and 3, which were not different (*P* > 0.05) from each other. The 50kW (3,223 kg/ha) solar sites had less (*P* < 0.05) biomass compared with the 30kW solar site (8,968 kg/ha) and the CON pasture (9,987 kg/ha) due to the lack of solar radiation reaching the plant-level (Laub et al., 2022). However, light penetration was not measured in the current study. The alfalfa, meadow fescue, orchardgrass, red clover, white clover, and grass legume mixes had similar biomass amounts for the 30kW site compared with the CON pasture (Figure 1). Alfalfa, red clover, and white clover were lower (*P* < 0.05) for the 50kW site compared with CON. Similarly, previous literature reported forage crops in AV sites to yield at least 90% of the control or even greater than the control during a drought year, such as the 2022 growing season in the current study (Amaducci et al., 2018; Trommsdorff et al., 2021; Jo et al., 2022). These similar biomass values concur with past modeling which predicted lower value forage crops to be a more profitable option in an AV grazing system (Feuerbacher et al., 2021). The sorghum-Sudangrass had the greatest biomass in the control pasture and 30kW sites and all sites were different from each other (Figure 1). However, the sorghum-Sudangrass had less (*P* < 0.05) biomass in the 50kW compared with other forages. Sorghum-Sudangrass is a summer annual grass that requires greater sun exposure compared with cool-season perennial grasses such as orchardgrass and meadow fescue (Gelley et al., 2016; DeBoer et al., 2017). Because the CON, 30kW, and 50kW treatments were associated with preexisting field locations, treatment effects may be partially confounded with site-specific effects such as soil variability. Although all locations were on the same farm and in close proximity, these effects cannot be completely excluded.

**Figure 1:**
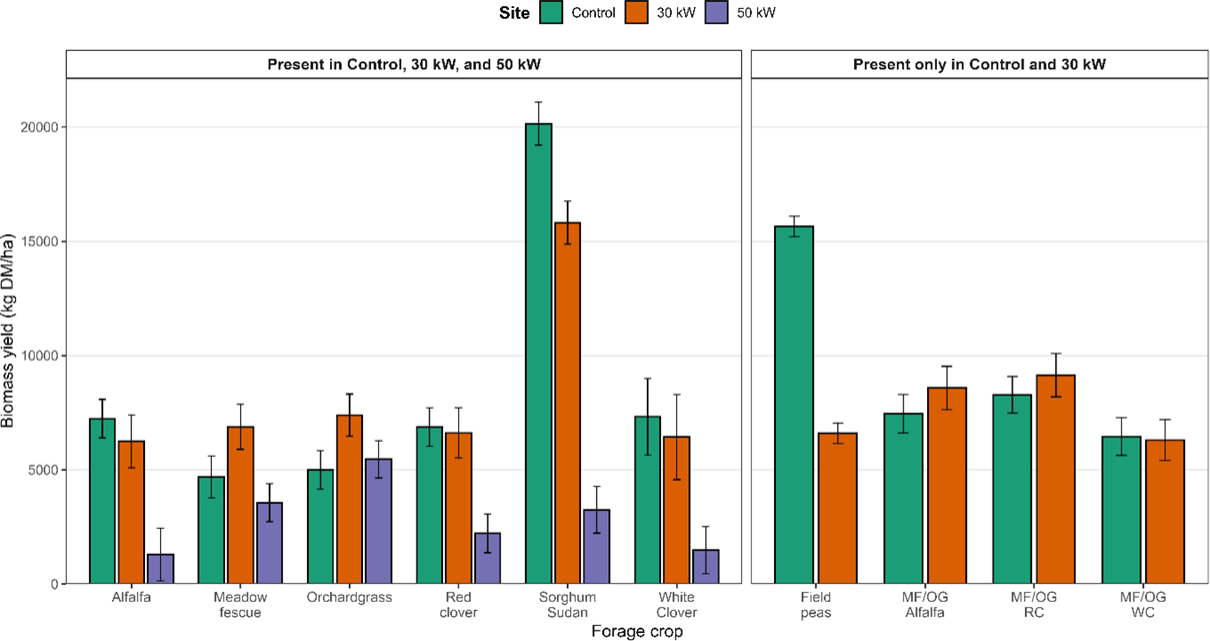
Forage biomass yield (kg DM/ha) of grasses and legumes grown under control pasture (control), 30-kW, and 50-kW agrivoltaic systems during 2022 and 2023. Values represent least squares means averaged across years and cuttings (n = 4 replicate rows per treatment). Error bars indicate SEM. MF = meadow fescue; OG = orchardgrass; RC = red clover; WC = white clover.

When comparing the 2 AV sites, 50kW had less (*P* < 0.05) biomass compared with 30kW for alfalfa, meadow fescue, red clover, and sorghum-Sudangrass (Figure 1). The 50kW site has had a larger area under shade, and research has shown that greater shade reduces weed pressure in forage production systems due to the lack of sunlight (Krieger et al., 2022; Marques et al., 2022; Wang et al., 2022). The alfalfa, red clover, and white clover produced drastically less biomass across all sites compared with previous studies conducted in Minnesota without solar panels (Catalano et al., 2019; Cherney et al., 2020). These differences may partially be a result of the 2022 drought conditions. White clover is particularly sensitive to drought, and this may explain the low biomass yield observed (Kanneganti et al., 1998). Low biomass production may also be a result of high weed pressure due to organic management or the low seeding rate for white clover (Schipanski et al., 2017).

Dry matter percentage on average was highest (*P* < 0.05) for the CON forages (25.1%), followed by the 30kW forages (16.8%) and the 50kW forages (12.8%). Differences in DM may have been affected by greater soil moisture retention, lower air temperatures, and lower vapor pressure deficit in the AV sites (Sturchio et al., 2022; Zhang et al., 2023). Increased shade in the AV sites likely altered microclimatic conditions, including soil temperature and evapotranspiration, which may have influenced soil moisture dynamics; however, these variables were not directly measured in the current study and are inferred from previous AV research (Marrou et al., 2013; Amaducci et al., 2018; Williams et al., 2023). Dry matter variations may be a result of differing stages of forage maturity across sites at the time of harvest; however, all forages were in the vegetative stage similar to Allen et al., (2013). Additionally, the 30kW and 50kW forages have greater NDF and ADF concentrations suggesting they were not at an earlier maturity stage compared with CON forages as ADF and NDF levels increase with maturity (Karn et al., 2006; Gelley et al., 2016).

The first cutting for all forages had more biomass (*P* < 0.05) compared with cuttings 2 and 3, which were not different (*P* > 0.05) from each other. Average CP for forages (Figure 2) in the 50kW (23.8% of DM) was higher (*P* < 0.05) than the 30kW (20.1% of DM) and CON (18.2% of DM). Crude protein has previously been found to be negatively correlated with forage biomass, which agrees with the higher CP values and lower forage biomass of the AV sites (Gelley et al., 2016). A similar trend is noted for individual species (Figure 2). The CP levels of the orchardgrass and meadow fescue for all 3 sites were higher than previous literature without solar panels (Karn et al., 2006; Allen et al., 2013). Comparison of CP across studies may be challenging because of differences in species composition, climate, soil conditions, management practices, and stage of maturity at harvest. In the current study, forages grown in the AV sites generally had greater CP compared with the CON, a pattern consistent with previous research reporting increased forage CP under reduced light or different climatic conditions (Catalano et al., 2019). For the current study, sorghum-Sudangrass grown under the 50kW system had higher CP than under the 30kW and CON pasture. This is similar to previous research that showed an inverse relationship between biomass accumulation and CP, because CP declines with advancing maturity of forages (Gelley et al., 2016). Reduced radiation under the 50kW array may have slowed forage growth and limited fiber accumulation, which may have contributed to greater CP. Higher CP levels may also be due to the 2022 drought conditions which can produce forages with higher nutritive values (Deetz et al., 1996). The field peas in the CON had lower CP and the field peas in the 30kW had similar CP to previous research studies (Greveniotis et al., 2022). Overall, prior AV studies documented increases in CP for various crops grown under solar panels compared with pasture controls (Thompson et al., 2020; Andrew et al., 2021; Kampherbeek et al., 2023).

**Figure 2:**
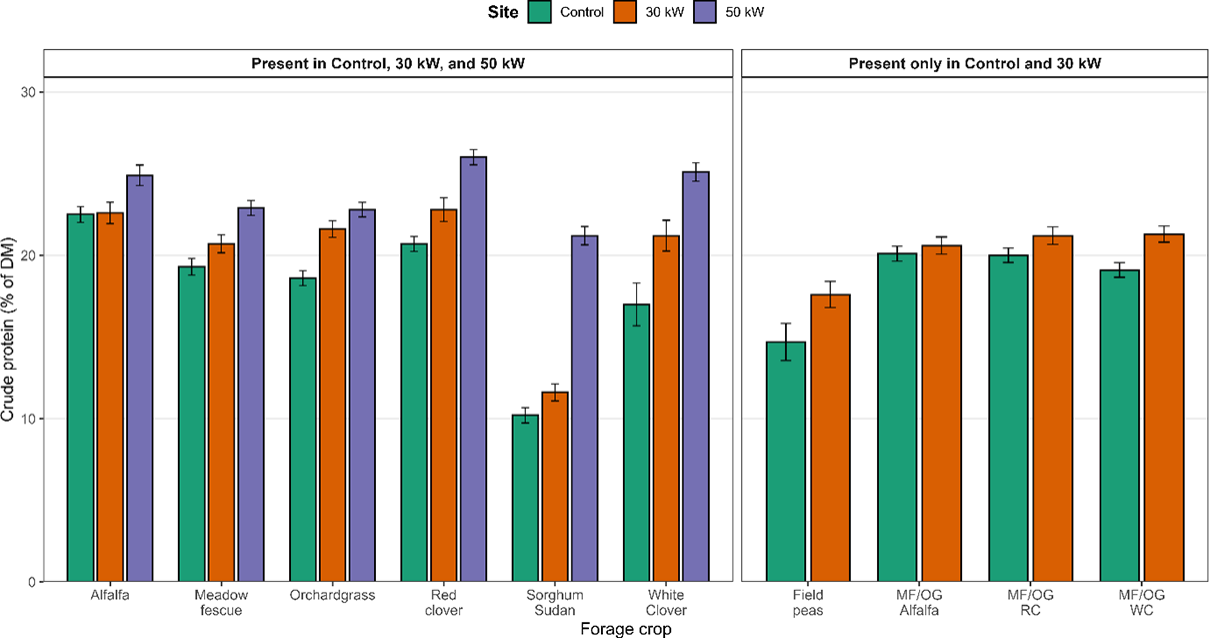
Crude protein concentration (% of DM) of grasses and legumes grown under control pasture (control), 30-kW, and 50-kW agrivoltaic systems during 2022 and 2023. Values represent least squares means averaged across years and cuttings (n = 4 replicate rows per treatment). Error bars indicate SEM.

The digestibility of NDF was measured by the TTNDFD (Figure 3). The CON (49.1%) and 30kW (52.3%) had similar TTNDFD, whereas the 50kW (54.4% of) had higher (*P* < 0.05) digestibility of forages. Neutral detergent fiber digestibility has previously been found to be negatively correlated with forage biomass, which is in agreement with the values reported in this study (Gelley et al., 2016). The forages with the most highly digestible TTNDFD were the sorghum-Sudangrass and orchardgrass in the 30kW, the white clover and sorghum-Sudangrass in the 50kW, and the meadow fescue and sorghum-Sudangrass in the CON (Figure 3).

**Figure 3:**
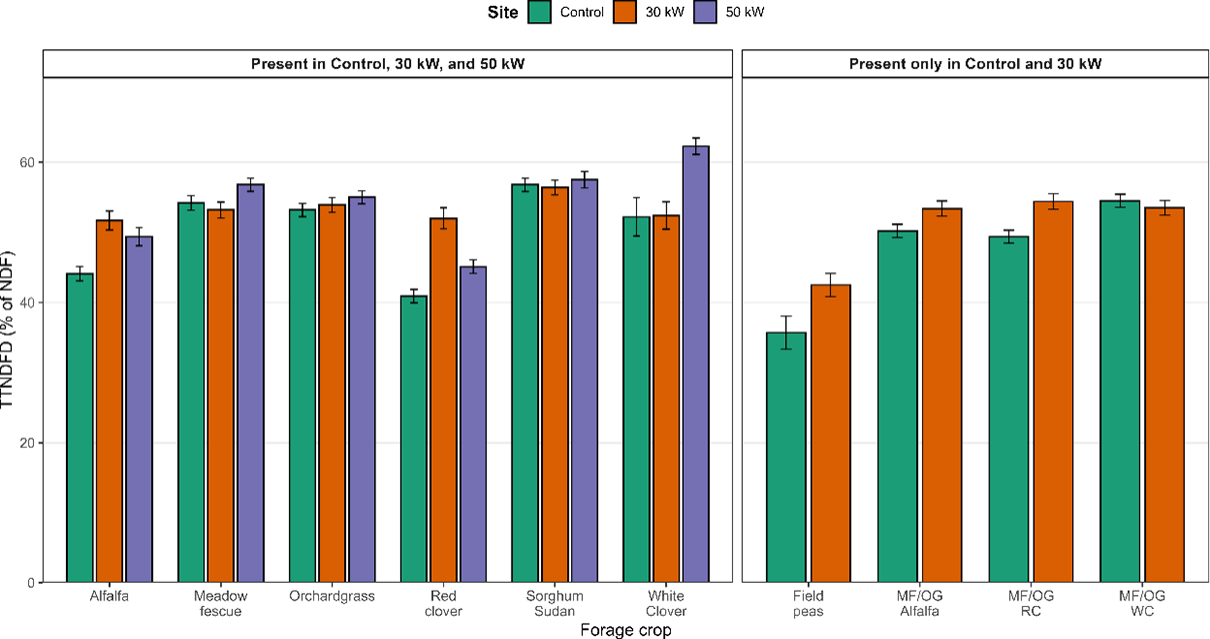
Total-tract NDF digestibility (TTNDFD; % of NDF) of grasses and legumes grown under control pasture (control), 30-kW, and 50-kW agrivoltaic systems during 2022 and 2023. Values represent least squares means averaged across years and cuttings (n = 4 replicate rows per treatment). Error bars indicate SEM.

Mineral content was also analyzed for forages for each site. The CON (0.796% of DM) and 30kW (0.759% of DM) had similar forage calcium (Ca) levels whereas the 50kW (1.06% of DM) forages had higher (*P* < 0.05) Ca content. The forages with the highest Ca levels were the red clover and field peas in the 30kW and CON pasture and the red clover and alfalfa in the 50kW. The forages at the 3 sites had (*P* < 0.05) different average phosphorus (P) levels with the CON forages (0.33% of DM) the lowest, the 30kW forages (0.39% of DM) intermediate and the 50kW forages (0.46% of DM) the highest. The forages with the highest P levels were the field peas and meadow fescue in the 30kW, the sorghum-Sudangrass and alfalfa in the 50kW, and the meadow fescue in the CON. The P content of 30kW alfalfa was similar to a previous study without PV, whereas the 50kW alfalfa had greater P levels and the CON alfalfa had lower P levels (Catalano et al., 2019). The sorghum-Sudangrass P levels in the 30kW site were similar to the vegetative and mature stages when grown without solar panels, whereas the CON P levels were lower and 50kW P were levels higher (DeBoer et al., 2017).

Species responses to AV shading differed substantially, indicating that forage selection is an important consideration for grazing systems integrated with photovoltaic arrays. Cool-season perennial grasses such as meadow fescue and orchardgrass had stable biomass production under moderate shading while providing acceptable nutritive value. Legumes, particularly red clover, consistently had high CP and forage quality across treatments, suggesting good adaptability to reduced-light environments. Conversely, the warm-season annual sorghum-Sudangrass had reduced biomass under the heavily shaded 50kW array, indicating that species requiring high light intensity may be less suitable beneath dense panel AV configurations. These results suggest that cool-season perennial grass and legume mixtures, particularly those including red clover, may represent the most reliable forage option for AV grazing systems where both biomass production and forage nutritive value are desired. Selection of shade-tolerant perennial species may be more important than maximizing forage biomass when designing pasture systems beneath solar arrays.

Although biomass production declined under greater shading in the AV systems, forage nutritive value was maintained or improved, as indicated by similar or higher CP, fiber digestibility, and mineral content in the 30kW and 50kW treatments compared with the CON pasture. Further documentation of microclimatic conditions in AV sites will help describe mechanisms for these differences in forage production for dairy grazing systems. Direct measurement of soil microclimate variables (e.g., soil moisture and temperature) should be included in future AV grazing studies to better quantify the mechanisms driving forage responses. Agrivoltaics in the form of forage production grown underneath ground-mounted photovoltaic systems can provide a suitable feed source for grazing livestock production, a green energy source for farms and additional income for farmers.
